# Exploratory study data for determining the adverse effects of sinomenine plus gabapentin or ligustrazine hydrochloride and the pharmacokinetic insights of sinomenine in plasma and CNS tissue

**DOI:** 10.1016/j.dib.2019.104633

**Published:** 2019-10-08

**Authors:** Tianle Gao, Tao Li, Wei Jiang, Weiming Fan, Jian-Dong Jiang

**Affiliations:** a State Key Laboratory of Bioactive Substances and Function of Natural Medicine, Institute of Materia Medica, Chinese Academy of Medical Sciences, Beijing 100050, China; b Beijing Key Laboratory of Traditional Chinese Medicine Basic Research on Prevention and Treatment of Major Diseases, Experimental Research Center, China Academy of Chinese Medical Sciences, Beijing 100700, China; c Zhejiang Zhenyuan Pharmaceutical Co., Ltd, 1015 West Shengli Road, Shaoxing, Zhejiang 312000, China

**Keywords:** Sinomenine, Ligustrazine hydrochloride, Drug combination, Adverse effects, Pharmacokinetics

## Abstract

This paper contains data that can be used in interpretation of the pharmacological effects of sinomenine combined with gabapentin or ligustrazine hydrochloride on chronic pain. The data can be divided into two parts. The first part is regarding if there were noticeable side effects accompanying drug applications of sinomenine plus gabapentin or ligustrazine hydrochloride. These side effects include sedation and change of core body temperature as well as tissue edema and sustained itch. The data were acquired from the open field test in mice, and provided insights for the effects of drug combination therapy on locomotive activities, rearing behaviors and body temperature. The second part is regarding whether sinomenine could be accumulated in the central nervous system (CNS) tissue following repeated drug administration. The data were acquired using microdialysis, which illustrated the pharmacokinetic properties of sinomenine, by showing relative concentrations of sinomenine in blood and CNS tissue, following single or repeated drug application. Data presented here is related to and supportive of the research article by Gao et al., “Sinomenine facilitates the efficacy of gabapentin or ligustrazine hydrochloride in animal models of neuropathic pain”[1], where interpretation of the research data presented here is available.

**Table:** Specifications Table

Subject area	Biology
More specific subject area	Pharmacology, Pain
Type of data	Figure
How data was acquired	Behavioral testing in animals with manual quantification. LC-20AC HPLC system coupled to a triple quad 6500 + mass spectrometer (HPLC-QqQ-MS) equipped with a Turbo V ion source (AB Sciex, Singapore) was used for pharmacokinetics. Analyst™ 1.7.1 software (Applied Biosystems, Foster City, CA) was applied to data collection, processing, and analysis.
Data format	Raw and analyzed
Experimental factors	The blood microdialysis probe is with 4 mm effective membrane length; 20,000 molecular weight cut-off and was positioned within the jugular vein toward the right atrium and then perfused with anti-coagulant citrate dextrose solution consisting of citric acid 3.5 mM, sodium citrate 7.5 mM, and dextrose 13.6 mM. The brain microdialysis probe was implanted in the corpus striatum zone and perfused with Ringer's solution (consisting of NaCl 145.3 mM; KCl 4.01mM; CaCl2 2.97 mM; pH 7.0) at 1.5 μl/min.
Experimental features	For assessment of potential side effects, the total travel distance, number of rearing behaviors, the duration of passivity and rectal temperature were recorded.
Data source location	Beijing, China.
Data accessibility	The data was attached with this article.
Related research article	T. Gao, T. Shi, Z. Wiesenfeld-Hallin, T. Li, J.-D. Jiang, X.- J. Xu, 2019. Sinomenine facilitates the efficacy of gabapentin or ligustrazine hydrochloride in animal models of neuropathic pain. Eur. J. Pharmacol., 854,101–108. https://doi.org/10.1016/j.ejphar.2019.03.061 [1].

**Table:** 

**Value of the Data** • Data presented in this article is helpful for understanding the potential side effects of sinomenine plus gabapentin or ligustrazine hydrochloride in animals. • Data presented in this article could provide pharmacokinetic insights of sinomenine in plasma and CNS tissue after intravenous injection. • Data presented in this article could be used to understand the underlying mechanisms of sinomenine drug combinations in chronic pain management.

## 1 Data

**Fig. 1:**
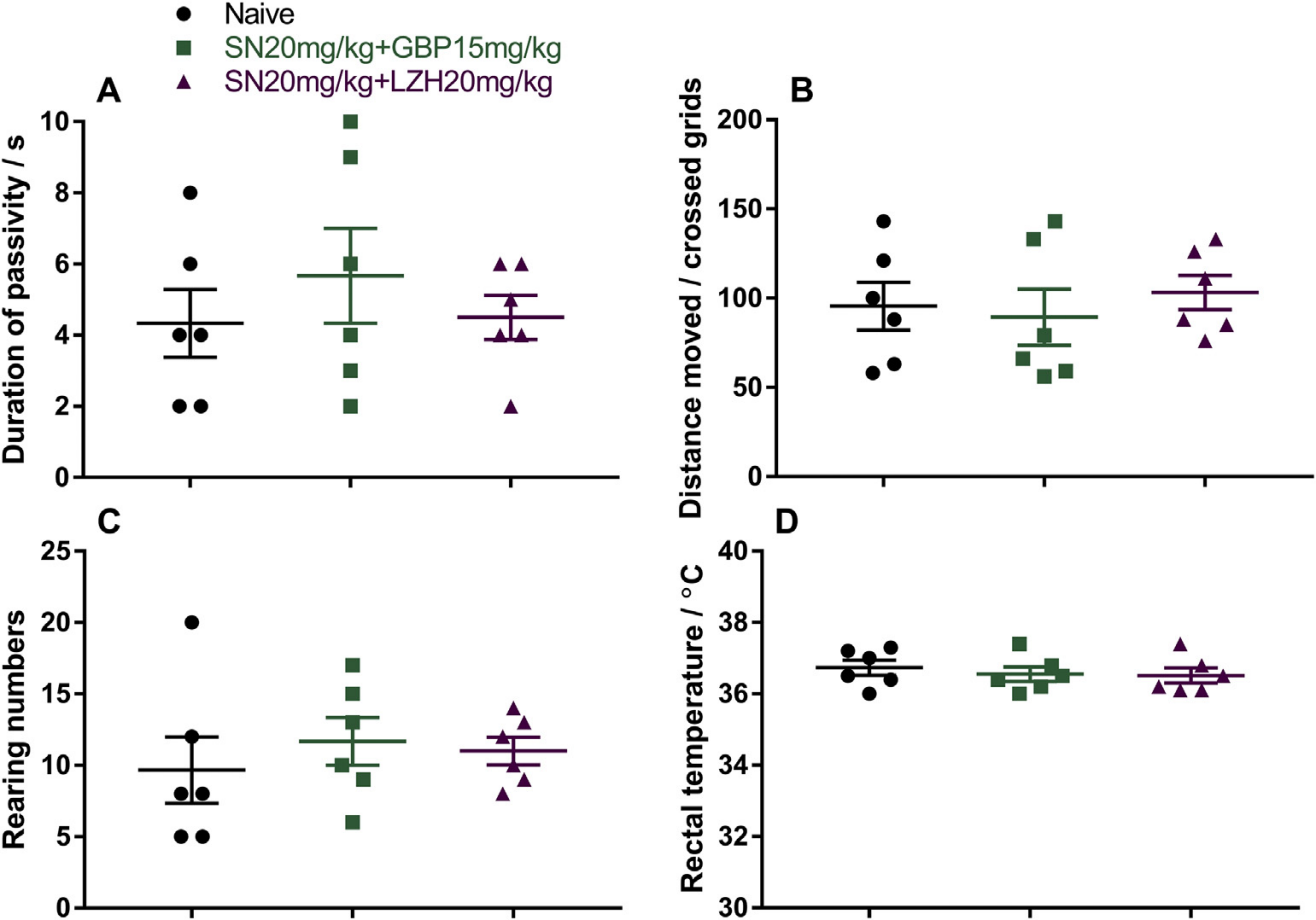
To investigate whether sinomenine (SN) combined with gabapentin (GBP) or ligustrazine hydrochloride (LZH) could generate noticeable adverse effects, mice were i.p. treated with “SN20mg/kg + GBP15mg/kg”, or “SN20mg/kg + LZH20mg/kg”, duration of passivity (A), locomotion (moved distance, B), numbers of rearing behaviors (C), and rectal temperatures (D) were quantified and compared with naïve mice without any pharmacological or vehicle treatment. N = 6 mice for each group. Data was presented as Mean ± SEM. One-Way ANOVA indicated that there were no significant differences between the groups. No significant differences were detected between naïve and drug-treated mice. No sign of tissue edema or sustained itch was discovered in association with drug application.

**Fig. 2:**
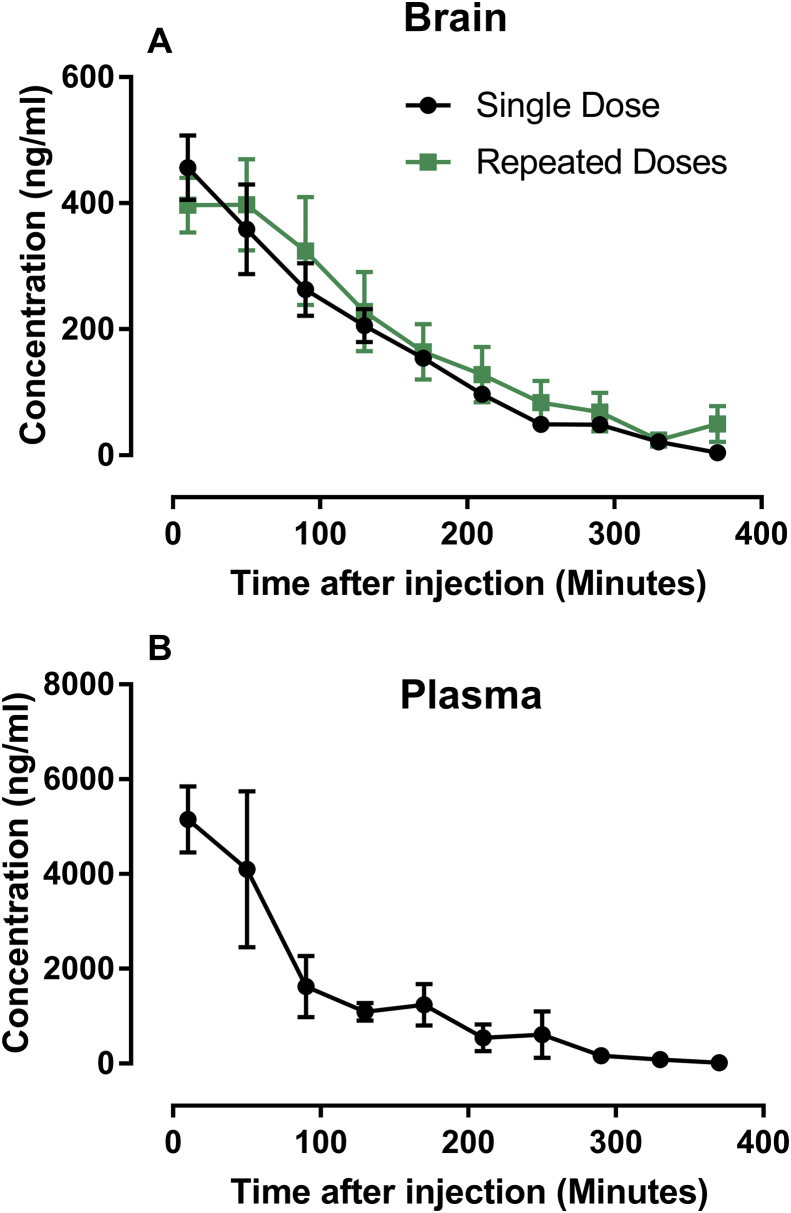
To study if pharmacokinetics of sinominine (SN) in plasma and brain share similar characteristics, we continuously collected samples (using microdialysis), and detected SN concentrations in striatum of the brain (extracellular levels, A) and the blood (B). N = 2–3 rats for each group. Data was presented as Mean ± SEM. Following SN (50mg/kg, intravenous) single or repeated administrations (once per day for 3 days, detecting samples were collected after last injection at day 3). Under single SN administration, SN concentrations was higher in blood than in brain. Also, SN concentrations in blood and brain followed similar trend, which was illustrated by sharp reduction and almost completely cleared in around 6 h from 5150.65 ± 1212.09 (Mean ± SD) ng/ml at 0 h to 17.72 ± 12.53 (Mean ± SD) ng/ml at 6 h in plasma; and from 456.63 ± 88.48 (Mean ± SD) ng/ml at 0 h to 4.15 ± 2.07 (Mean ± SD) ng/ml at 6 h in extracellular striatal tissue; SN concentrations at 6 h in plasma or extracellular striatal tissue are considered as ultralow levels). Following repeated SN administration, SN concentrations resembled the trend of the single dose application (green square versus black circle, A), with SN concentrations sharply reduced from 397.17 ± 61.97 (Mean ± SD) ng/ml at 0 h to 49.79 ± 40.01 (Mean ± SD) ng/ml at 6 h in extracellular striatal tissue. The data showed that sinomenine could be cleared in around 6 h after drug application, and even there is uncleared drugs remained before first drug application at each day, the concentrations should be at ultralow level. There is no chance to generate drug accumulation of SN by repeated dosing especially in the targeted tissue (Brian), which is confirmed by testing SN's brain concentration under repeated dosages (A). The elevated baseline discovered in our pharmacological study [1], is more likely to be related to the pharmacodynamics effects than pharmacokinetics effects of the repeated drug combination.

In our earlier published data, the repeated drug administration of sinomenine 10mg/kg plus gabapentin 4mg/kg reduced baseline hypersensitivity, and resulted in persistent reduction in allodynia with increased baseline thresholds [1]. Here we report experimental data on if there were severe side effects accompanying drug applications of sinomenine plus gabapentin or ligustrazine hydrochloride that could affect the rodent's behaviors, or severe allergy that could result in the reduction of body temperature. Shown in Fig. 1 (Table 1 in Raw Dataset), duration of passivity (Fig. 1A), locomotion (moved distance, Fig. 1B), numbers of rearing behaviors (Fig. 1C), and rectal temperatures (Fig. 1D) were recorded after applications of drug combinations and compared with naïve mice. We have earlier discovered after repeated administration of sinomenine plus gabapentin, the baseline pain threshold was increased [1]. We know that the interchange of gabapentin between blood and CNS tissue is occurring at an extremely fast speed, however there is lack of relevant data for the interchange rate of sinomenine between blood and CNS tissue, to understand whether sinomenine could be accumulated in CNS tissue at pharmacologically active doses and intervals. To explain if the increased baseline pain threshold under repeated administration of sinomenine plus gabapentin [1] is associated with accumulation of sinomenine, we acquired additional data (Fig. 2, Table 2 in Raw Dataset). Using microdialysis technique, Data in Fig. 2 illustrated the changes of sinomenine concentration in CNS tissue (Fig. 2A) and plasma (Fig. 2B) after single or repeated injections. Post-drug concentrations of sinomenine in CNS tissue followed similar trend under single or repeated injections in rats (Fig. 2A), which provided evidence that the increased baseline drug efficacy seen in our earlier published data [1] was unlikely due to an accumulation of the drug following repeated injections.

## 2 Experimental design, materials and methods

### 2.1 Experimental design

Previously we have found sinomenine can be used for the treatment of chronic pain in animal models [[2], [3], [4]]. Recently, we were able to show that there are additional benefits by combining sinomenine with gabapentin or ligustrazine chloride in treating neuropathic pain situation [1]. In order to support this finding and show that there were no noticeable side effects accompanying drug applications of sinomenine plus gabapentin or ligustrazine hydrochloride, we performed open filed test in mice applied with above mentioned drug combinations, which was shown in data section in Fig. 1. To further give supportive data to understand whether the elevation of baseline pain threshold after repeated administration of sinomenine plus gabapentin, is related to accumulation of each drugs in CNS tissue, we performed additional tests using 2–3 rats with single or repeated (once per day for 3 days) intravenous injection of sinomenine at 50mg/kg, which could illustrate sinomenine's pharmacokinetics difference in plasma and in CNS tissue. This data was shown in Fig. 2. Detailed materials and methods of the presented data are mentioned below.

### 2.2 Animals

All mice (C57BL/6, male, from Beijing Vital River Laboratory Animal Technology, China) or rats (SD, male, from Beijing Vital River Laboratory Animal Technology, China), background information for C57BL/6 mice and SD rats can be found in Raw Dataset Table.3. Animals were age matched and the littermates were housed together in the same cage, with 6 per cage for mice or 4 per cage for rats respectively, in a standard laboratory condition (22 °C 12 h light/dark cycle) with ad libitum access to water and food. For Fig. 1 and Raw Dataset Table.1, “SN20mg/kg + GBP15mg/kg”, “SN20mg/kg + LZH20mg/k” or “Naïve” group were comprised by randomly combining 2 mice from each of 3 age matched cages (6 mice per cage). For Fig. 2 and Raw Dataset Table.2, rats received single or repeated sinomenine injection were selected from 2 different age matched cages (4 rats per cage).

### 2.3 Assessment of side effects

To assess if drug combinations induce motor deficiencies, sedation or change of body temperature in mice, we performed an open field test. The open field arena is 50 cm × 50 cm with 25 grids (the area of one grid is 10 cm × 10 cm), in which mice (without any previous experience in the open field test) were allowed to move freely for 5 min. As illustrated in our previous published study [3], the total travel distance (quantified by the number of passed grids), number of rearing behaviors and duration of passivity (time when animal showed no movement) were recorded and quantified manually, and rectal temperature was recorded using a rodent thermometer (Bioseb, USA).

### 2.4 Pharmacokinetics study using microdialysis

Pharmacokinetics study using microdialysis was performed in SD rats. The blood microdialysis probe (4 mm effective membrane length; 20,000 molecular weight cut-off; CMA Microdialysis, Sweden) was positioned within the jugular vein toward the right atrium and then perfused with anti-coagulant citrate dextrose solution consisting of citric acid 3.5 mM, sodium citrate 7.5 mM, and dextrose 13.6 mM. The brain microdialysis probe was implanted in the corpus striatum zone and perfused with Ringer's solution (consisting of NaCl 145.3 mM; KCl 4.01mM; CaCl2 2.97 mM; pH 7.0) at 1.5 μl/min after a stabilization period of 1 h post probe implantation. After intravenous drug administration of sinomenine at 50 mg/kg, simultaneous collection of microdialysis samples in blood and striatum of brain were performed at different time points (20 min/sample, collecting for at least 6 h). An established high performance liquid chromatography coupled with triple quadrupole mass spectrometry method [5], was deployed for detection of sinomenine concentrations, and the results were corrected with in vitro recovery of the probe.

### 2.5 Drugs

For preparation of sinomenine injection solution, sinomenine (CAS No. 6080-33-7, Catalog No. 110774, obtained from the National Institute for Food and Drug Control, Beijing, China, purity >99%) was dissolved in DMSO (CAS No. 67-68-5, Catalog No. 34869, obtained from Sigma-Aldrich, Germany, purity >99%), then mixed with Cremophor EL oil (CAS No. 61791-12-6, Catalog No. 238470, Sigma-Aldrich, Germany) and saline by vortex mixer (Bibby Scientific, UK) using the volume rate of 1:4:5. Any further dilution was mixed with saline (CAS No. 7647-14-5, Catalog No. S0817, obtained from Sigma-Aldrich, Germany). For preparation of gabapentin injection solution, gabapentin (CAS No. 60142-96-3, Catalog No. 1287303, obtained from obtained from Sigma-Aldrich Research Biochemicals Inc., USA) was dissolved in saline. Vehicle for gabapentin was saline solution. For preparation of ligustrazine hydrochloride injection solution, ligustrazine hydrochloride (CAS No. 76494-51-4, Catalog No. 3628, Sinova Inc., USA) was dissolved in saline.

### 2.6 Blinding

For experiment data in Fig. 1 and Raw Dataset Table.1, drugs are blinded by introducing Drug Codes (labeled by one person who does not perform the actual experiment), and decoded after data analysis. However, for experiment data in Fig. 2 and Raw Dataset Table.2, blinding was not used since there is only one condition/group for single or repeated drug application scheme.

### 2.7 Statistics

Data were presented as mean ± SEM. Bonferroni post hoc test following One-Way ANOVA were used to analyze the difference between groups, P < 0.05 was considered as statistically significant (Fig. 1, Raw Dataset Table.2). For Fig. 2 and Raw Dataset Table.2, considering that it is an exploratory approach to investigate the pharmacokinetics of single and repeatedly injected sinomenine, with only 2 to 3 animals per group, power analysis was not used.

## Institutional review board statement for ethics in animal study

All animal studies were performed at the animal facility of Experimental Research Center, China.

Academy of Chinese Medical Sciences, Beijing, China. Animal experiments were conducted following the National Guidelines for Housing and Care of Laboratory. Animals and performed in accordance with protocol approved by the Research Ethics Committee at China Academy of Chinese Medical Sciences, Beijing, China.

## References

[1] Gao T., Shi T., Wiesenfeld-Hallin Z., Li T., Jiang J.-D., Xu X.-J. (2019). Sinomenine facilitates the efficacy of gabapentin or ligustrazine hydrochloride in animal models of neuropathic pain. Eur. J. Pharmacol..

[2] Gao T., Hao J., Wiesenfeld-Hallin Z., Wang D.Q., Xu X.J. (2013). Analgesic effect of sinomenine in rodents after inflammation and nerve injury. Eur. J. Pharmacol..

[3] Gao T., Shi T., Wiesenfeld-Hallin Z., Svensson C.I., Xu X.-J. (2015). Sinomenine alleviates mechanical hypersensitivity in mice with experimentally-induced rheumatoid arthritis. Scand. J. Pain.

[4] Gao T., Shi T., Wang D.-Q., Wiesenfeld-Hallin Z., Xu X.-J. (2014). Repeated sinomenine administration alleviates chronic neuropathic pain-like behaviours in rodents without producing tolerance. Scand. J. Pain.

[5] Zhao X.-L., Li T., Liu Y., Zhang M.-Y., Chen Y., Cui Y., Zhang Y., Sun D.-D., Wang Z.-G., Wang D.-Q. (2015). Pharmacokinetic analysis of sinomenine based on automatic blood sampling system and HPLC-QQQ-MS. Chin J Exp Tradit Med Formul.

