# Supplementary material for: Exploratory study data for determining the adverse effects of sinomenine plus gabapentin or ligustrazine hydrochloride and the pharmacokinetic insights of sinomenine in plasma and CNS tissue
Source: Data Brief. 2019 Oct 8;27:104633. doi: 10.1016/j.dib.2019.104633 (PMC6812036; doi:10.1016/j.dib.2019.104633)
Supplement: Multimedia component 1 [file mmc1.docx]

| Duration of Passivity (second) | | |
| --- | --- | --- |
| Naïve | **Sinomenine 20mg/kg + Gabapentin 5mg/kg** | **Sinomenine20mg/kg + Ligustrazine Hydrochloride 20mg/kg** |
| 8 | 9 | 4 |
| 4 | 3 | 6 |
| 6 | 10 | 6 |
| 2 | 6 | 5 |
| 4 | 4 | 2 |
| 2 | 2 | 4 |
| Distance moved (crossed grids) | | |
| Naïve | **Sinomenine 20mg/kg + Gabapentin 5mg/kg** | **Sinomenine 20mg/kg + Ligustrazine Hydrochloride 20mg/kg** |
| 88 | 79 | 88 |
| 143 | 56 | 111 |
| 100 | 133 | 126 |
| 121 | 66 | 76 |
| 63 | 59 | 133 |
| 58 | 143 | 85 |
| Rearing numbers | | |
| Naïve | **Sinomenine 20mg/kg + Gabapentin 5mg/kg** | **Sinomenine 20mg/kg + Ligustrazine Hydrochloride 20mg/kg** |
| 8 | 15 | 10 |
| 12 | 13 | 13 |
| 5 | 6 | 12 |
| 5 | 10 | 9 |
| 20 | 9 | 14 |
| 8 | 17 | 8 |
| Rectal Temperature (°C) | | |
| Naïve | **Sinomenine 20mg/kg + Gabapentin 5mg/kg** | **Sinomenine 20mg/kg + Ligustrazine Hydrochloride 20mg/kg** |
| 36 | 36.5 | 36.8 |
| 37.2 | 36 | 37.4 |
| 37.3 | 36.8 | 36.1 |
| 36.4 | 37.4 | 36.2 |
| 36.5 | 36.2 | 36.1 |
| 37 | 36.4 | 36.5 |

**Table 1. Raw data for Figure 1, * No sign of tissue edema or sustained itch was discovered in association with drug application.**

| Plasma concentration of Sinomenine (ng/mL) following single dose Sinomenine application at 50 mg/kg | | | | | | | |
| --- | --- | --- | --- | --- | --- | --- | --- |
| Tim（h） | **No.1#** | | **No.2#** | | **No.3#** | **Mean ± SD** | |
| 0 | 4174.83 | | 4769.65 | | 6507.46 | 5150.65±1212.09 | |
| 0.67 | 3082.83 | | 1904.64 | | 7315.3 | 4100.92±2845.38 | |
| 1.33 | 1948.01 | | 388.36 | | 2543.84 | 1626.74±1113.07 | |
| 2 | 990.62 | | 816.8 | | 1459.77 | 1089.06±332.6 | |
| 2.67 | 2110.91 | | 929.34 | | 677.09 | 1239.11±765.46 | |
| 3.33 | 1089.71 | | 166.41 | | 379.24 | 545.12±483.49 | |
| 4 | 1592.28 | | 93.03 | | 154.35 | 613.22±848.44 | |
| 4.67 | 389.13 | | 71.29 | | 48.24 | 169.55±190.51 | |
| 5.33 | 124.08 | | 47.27 | | - | 85.67±54.32 | |
| 6 | - | | 26.58 | | 8.86 | 17.72±12.53 | |
| Concentration of Sinomenine (ng/mL) in extracellular fluid in striatum following single dose sinomenine application at 50 mg/kg | | | | | | | |
| Tim（h） | **No.1#** | | **No.2#** | | **No.3#** | **Mean ± SD** | |
| 0 | 545.12 | | 456.63 | | 368.15 | 456.63±88.48 | |
| 0.67 | 270.85 | | 306.06 | | 498.97 | 358.63±122.81 | |
| 1.33 | 200.20 | | 246.88 | | 342.06 | 263.05±72.3 | |
| 2 | 159.86 | | 205.66 | | 251.46 | 205.66±45.8 | |
| 2.67 | 132.87 | | 154.24 | | 175.61 | 154.24±21.37 | |
| 3.33 | 98.82 | | 78.71 | | 113.41 | 96.98±17.43 | |
| 4 | 33.80 | | 47.11 | | 66.31 | 49.07±16.35 | |
| 4.67 | 58.27 | | 48.37 | | 38.47 | 48.37±9.90 | |
| 5.33 | 27.32 | | 14.57 | | 21.77 | 21.22±6.39 | |
| 6 | 4.15 | | 6.22 | | 2.08 | 4.15±2.07 | |
| Concentration of Sinomenine (ng/mL) in extracellular fluid in brain striatum following repeated sinomenine application at 50 mg/kg | | | | | | | |
| Tim（h） | | **No.4#** | | **No.5#** | | | **Mean ± SD** |
| 0 | | 353.35 | | 440.99 | | | 397.17±61.97 |
| 0.67 | | 469.49 | | 325.23 | | | 397.36±102.01 |
| 1.33 | | 410.39 | | 238.4 | | | 324.4±121.61 |
| 2 | | 290.85 | | 164.86 | | | 227.85±89.09 |
| 2.67 | | 208.19 | | 120.52 | | | 164.36±62 |
| 3.33 | | 172.19 | | 83.6 | | | 127.89±62.64 |
| 4 | | 118.51 | | 49.17 | | | 83.84±49.03 |
| 4.67 | | 99.16 | | 37.91 | | | 68.53±43.31 |
| 5.33 | | - | | 23.95 | | | 23.95±0 |
| 6 | | 78.08 | | 21.5 | | | 49.79±40.01 |

**Table 2. Raw data for Figure 2, “-” indicates missing data points based on technical failure in sample collection.**

| The C57BL/6 strain used in this article is from Beijing Vital River Laboratory Animal Technology, China, with following history: |
| --- |
| In 1921, C.C. Little bred the Miss Abby Lathrop strain (C57BR and C57L also derived from this strain). |
| In 1937, the 6th and 10th strains were separated. |
| In 1948, Jackson Laboratory introduced the 6th strain system from Hall. |
| In 1951, NIH introduced the 32nd generation of this line from Jackson Laboratory. |
| Charles River was introduced from NIH in 1974 and had a caesarean section in 1975. |
| In 2001, Vital River introduced the 59th generation core group from Charles River. |
| In 2007, Vital River conducted Cloneback on this line and re-introduced the 76th generation core group from Charles River. |
| In 2014, Vital River again introduced the 83rd generation core group from Charles River to Cloneback the line. |
| The SD strain used in this article is from Beijing Vital River Laboratory Animal Technology, China, with following history: |
| In 1925, Robert W. Dawley mate a hybrid male and a female Wistar rat to obtain the strain. |
| Charles River Laboratories introduced the line from Sprague Dawley Inc. in 1950 to form Charles River SDTM. |
| Caesarean section was conducted in 1955 and generated the CD core group. |
| In 1991, 8 populations were selected to form the IGS (International Genetics Standard) basic population. |
| Caesarean section was used to obtain a segregator population in 1997, named Crl: CD (SD) IGS (IGS: Laboratory Animal Breeding in Accordance with Charles River International Genetics Standard). |
| In 1999, Vital River introduced the core group of this line from Charles River. |
| In 2004, 2011 and 2014, in order to maintain the global unification of CD population traits, Vital River re-introduced the core group of the strain three times. |
| In 2016, Vital River introduced and updated the core group from Charles River. |

**Table 3. Background of C57BL/6 Strain (mouse) and SD Strain (rat) from Vital River**
